# Verbal Working Memory but Not Attention Is Related to Language Proficiency: Evidence from Multilingual Speakers

**DOI:** 10.5334/pb.525

**Published:** 2020-09-04

**Authors:** Marion Bouffier, Cristina Barbu, Steve Majerus

**Affiliations:** 1 University of Liège, Liège, BE; 2 Fund for Scientific Research – F.R.S.-FNRS, Brussels, BE

**Keywords:** multilingualism, language proficiency, early adulthood, auditory-verbal working memory, auditory-verbal attention, visuo-spatial attention

## Abstract

Numerous studies have shown a consistent relationship between verbal working memory (WM) and native-language as well as non-native language learning abilities. However, the role of attentional abilities has been rarely explored, although these abilities have been shown to be associated both with verbal working memory and oral language proficiency. This study investigated the association between WM, attention and language proficiency in young adults raised with three different languages (Luxembourgish, German and French). Auditory-verbal WM abilities were assessed via an immediate serial recall task. Attentional abilities were assessed via auditory-verbal and visuo-spatial attentional tasks. Using a Bayesian correlational approach, we observed robust evidence for an association between auditory-verbal WM abilities and non-native language proficiency. At the same time, we observed no reliable evidence in favor of an association between language proficiency and auditory-verbal/visuo-spatial attentional measures. These results suggest that auditory-verbal WM and non-native language proficiency are strongly linked in young multilingual adults, irrespective of auditory-verbal or visuo-spatial attentional abilities.

## Introduction

Verbal working memory (WM) is defined as the ability to temporarily store verbal information, with or without further manipulation of this information, in order to fulfill cognitive tasks such as language processing or reasoning (Baddeley, 1992). This ability has been considered to also play a crucial role in the acquisition of new verbal information, such as during native and second language learning (e.g., Baddeley, Gathercole, & Papagno, 1998). A large number of studies have shown reliable associations between verbal WM and lexical learning abilities as involved in native language or foreign language vocabulary learning (e.g., Gathercole, Service, Hitch, Adams, & Martin, 1999; Gathercole, Willis, Baddeley, & Emslie, 1994; Leclercq & Majerus, 2010; Majerus, Poncelet, Van der Linden, & Weekes, 2008; Service, 1992). At the same time, verbal working memory abilities are dependent upon auditory attention abilities, the latter also potentially being involved in language learning (e.g., Majerus, Heiligenstein, Gautherot, Poncelet, & Van der Linden, 2009). The aim of this study is to advance our understanding of the links between verbal WM, attention and the outcome of language learning; namely language proficiency, by examining the extent to which the link between verbal working memory and oral language proficiency reflects an association with attentional abilities, and this for both native and non-native language proficiency.

The association between verbal WM and language abilities has been demonstrated by studies relating verbal WM performance to measures of existing receptive vocabulary knowledge for native and foreign languages as well as to paired-associate word-novel word learning tasks mimicking novel vocabulary learning. In children, verbal working memory abilities as measured by nonword repetition tasks are positively associated with various language skills such as vocabulary, reading and sentence comprehension (Gathercole et al., 1994). Studies distinguishing between item (the linguistic identity of individual memoranda) and serial order (the order of presentation of memoranda) showed that it is not only the linguistic aspect of the memoranda (item information) that explains the association between verbal WM performance and language abilities, but also the need to maintain and recall the memoranda in correct serial order (Majerus et al., 2008; Majerus & Boukebza, 2013; Majerus, Poncelet, Elsen, & Van der Linden, 2006; Majerus, Poncelet, Greffe, & Van der Linden, 2006; Ordonez Magro, Attout, Majerus, & Szmalec, 2018). The latter findings indicate that the link between verbal WM and language measures cannot be only explained by the fact that both measures probe verbal levels of processing, but that serial order maintenance, a more specific aspect of verbal WM tasks, also exerts an important role (Leclercq & Majerus, 2010). These findings also support theoretical models of WM that distinguish linguistic components directly depending on the language system and specific serial order WM mechanisms (e.g., Burgess & Hitch, 1999, 2006; Hartley, Hurlstone, & Hitch, 2016; Majerus, 2019).

At the same time, verbal WM is not only relying on linguistic knowledge and serial order processing mechanisms, but has also been shown to interact with various aspects of attentional processing. At a general level, several models of WM lend a central role to attention (Baddeley, 1986; Barrouillet, Bernardin, & Camos, 2004; Cowan, 1995; Engle, Kane, & Tuholski, 1999). While in the WM framework proposed by Baddeley, executive attention abilities are supposed to intervene mainly when modality-specific short-term storage capacity limits have been reached (i.e., supra-span conditions), other authors such as Cowan consider that attention intervenes already at the most low-level short-term storage situations. According to Cowan’s embedded-processes model, memoranda are maintained in the focus of attention during WM tasks (Cowan, 2001, 2010). The focus of attention refers to the non-strategic attendance to memoranda (Cowan et al., 2005; Cowan, Fristoe, Elliott, Brunner, & Saults, 2006) and reflects the amount of verbal or visual information one is capable of holding in mind in the absence of any active maintenance processes such as rehearsal. This ability can be measured by running span tasks in which continuous stimulus sequences are presented at a very fast rate (two or three stimuli/second); presentation is then stopped at an unpredictable time and participants are requested to report the stimuli they still have in mind (Cowan et al., 2005; Kowialiewski & Majerus, 2018). Participants rarely report more than four items in these situations, which has led Cowan to propose that focus of attention capacity is limited to three to four items (Cowan, 2010). Another aspect of attention is the control of attention (Chow & Conway, 2015; Cowan et al., 2005, 2006), that is the top-down orientation of attention on specific stimuli as a function of current goals, whether generated internally or imposed from the outside (Corbetta & Shulman, 2002). In WM, controlled attention involves goal-related selection and, in specific contexts, manipulation of memoranda. This distinction between a more passive focus of attention capacity and more controlled attentional abilities is supported by interindividual difference studies (Cowan et al., 2005, 2006; Shipstead, Redick, Hicks, & Engle, 2012) as well as by neuroimaging studies (Majerus, Péters, Bouffier, Cowan, & Phillips, 2018). Critically for the research question of this study, these attentional abilities may also be involved in language learning and its outcomes. The perception and further processing of language stimuli, particularly if novel, is a challenging situation at the level of auditory-verbal attentional abilities (Gomes, Wolfson, & Halperin, 2007). Attentional limitations have been linked to difficulties in language acquisition (Montgomery, Evans, & Gillam, 2009). Moreover, in one of the few studies examining both the role of verbal WM and attentional abilities in language proficiency, Majerus et al. (2009) showed that auditory-verbal attention, as measured by a controlled attention task, explained a significant part of variance in native vocabulary abilities in six-to-seven-year-old children; this variance was partially shared with variance explained by verbal WM tasks. At the same time, the association between more ‘passive’ focus of attention abilities with either native or non-native language proficiency has not yet been explored.

On the other hand, it should also be noted that bilingual practice might have effects on cognition. In line with this hypothesis, some studies have shown that WM and other cognitive capacities can be enhanced in multilingual speakers as compared to monolingual speakers, for both verbal and non-verbal WM measures (Blom, Küntay, Messer, Verhagen, & Leseman, 2014; Delcenserie & Genesee, 2017; Papagno & Vallar, 1995). This finding has been termed the ‘bilingual cognitive advantage’ hypothesis (Adesope, Lavin, Thompson, & Ungerleider, 2010; Bialystok, 2009, 2011, 2015; Bialystok, Craik, Klein, & Viswanathan, 2004). However, the literature in this domain is far from being consistent. WM advantages in bilingual speakers have not been systematically replicated (Engel de Abreu, 2011; Lukasik et al., 2018). Luo, Craik, Moreno, and Bialystok (2013) even found a disadvantage in word-recall in bilingual adults. Antón, Carreiras, and Duñabeita (2019) found a bilingual advantage for verbal and non-verbal WM tasks, but only when the tasks had a high executive load such as backward digit span and the backward Corsi block-tapping test.

Similarly, some studies also suggest that attentional processes might be enhanced by bi- or multilingual practice. Some evoked-potential (ERP) studies tend to show that monolinguals and bilinguals allocate their attention to speech input in a different manner. Kuipers and Thierry (2015) showed that bilingual infants displayed an early positive deflection for matching versus non-matching verbal stimuli, whereas monolingual children did not display that deflection. Astheimer, Berkes, and Bialystok (2016) found larger responses during attentive speech processing in bilingual adults as compared to monolinguals. Furthermore, in a task where adult participants were asked to selectively listen to tones coming from only one of two streams, Rämä et al. (2018) found that bilinguals showed enhanced responses linked to maintenance of selective attention and to disengagement of attention from distracting auditory stimulation.

The aim of this exploratory study was to assess the interrelations between verbal WM, focus of attention, control of attention and language proficiency in adult, multilingual speakers educated in German (L2) and French (L3), and raised in Luxembourgish (L1). The use of multilingual participants allowed us to assess the interrelations between WM, attention and language abilities for both native and non-native language proficiency. Note that we focused in this study mainly on lexical levels of multilingual language proficiency. We hypothesized that the link between attentional and language abilities should be more pronounced for L2 and L3, the less proficient languages which require more attentive processing during perception and production.

A final specificity of this study was to assess focus and control of attention in both auditory-verbal and visuo-spatial modalities. Attentional abilities involved in WM have been claimed by a number of studies to be amodal (Baddeley, 1986; Cowan, 1995; Majerus et al., 2016) but not by others (Majerus et al., 2018; Morey & Cowan, 2005). In this study, we examined which aspect of attention, focus of attention versus control of attention, is associated with language proficiency and verbal WM, and whether the associations are the same for auditory-verbal versus visuo-spatial attentional abilities.

## The present study

The participants of this study were all native adult citizens of Luxembourg, a country in which the mother tongue is Luxembourgish, a language of the family of Germanic languages. All Luxembourgish citizens start learning German as L2 at first grade and French as L3 at second grade. They are under significant pressure to master German and French given that the teaching language for school subjects other than language classes (mathematics, sciences) is German, followed by French (the latter mostly during the secondary school curriculum). Furthermore, once adults, Luxembourgish citizens are on a daily basis confronted to German and French speaking situations either at the social, professional or administrative level. By selecting this group of participants, we were able to assess their language proficiency in each of the three languages, which can vary to a considerable extent between L2 and L3 languages. Language proficiency was assessed via receptive and productive vocabulary tasks as well as via lexical decision tasks. We calculated for each language a summary proficiency index which consisted of the mean of the standardized scores obtained in the different linguistic tasks.

WM abilities were assessed using a standard immediate serial recall task for verbal lists, but by using both familiar, word stimuli and unfamiliar, nonword stimuli (Majerus & Van der Linden, 2003). We used immediate serial recall tasks for assessing verbal WM as this study focused on the role of WM storage capacity on multilingual language proficiency. Most theoretical accounts linking WM to language learning abilities consider that it is the ability to temporarily maintain verbal items and their sequential arrangement (serial order) that is relevant for learning novel verbal information such as foreign word forms which are novel sequential arrangements of a limited set of phonemes. This aspect of WM is most directly measured by verbal immediate serial recall tasks in which lists of items have to be recalled immediately after presentation in correct serial order (Baddeley et al., 1998; Majerus et al., 2008; Majerus, Poncelet, Elsen, et al., 2006; Majerus, Poncelet, Greffe, et al., 2006). By probing verbal WM for both word and nonword stimuli, we were able to measure verbal WM abilities in a comprehensive manner, for the retention of both phonological and lexico-semantic levels of information. This task was exclusively administered in L1 in order to measure verbal WM abilities at their optimal level and without biasing performance due to variable levels of phonological and lexico-semantic knowledge for L2 and L3 stimuli.

Focus of attention abilities for auditory-verbal material were measured using the running span task procedure most commonly used for assessing this aspect (Cowan et al., 2005). Participants heard continuous sequences of alternating digits and letters at a speed of 2.5 items/second and had to focus on all stimuli as they appeared; the fast presentation speed prevented the implementation of controlled encoding strategies. When the list stopped, the participants had to recall all items they still had in mind. Control of attention for auditory-verbal material was assessed using an adaptation of the running span task (Majerus et al., 2018): participants were now instructed to focus only on the digits or the letters of the alternating digit-letter continuous sequence, requiring and enabling controlled selective attention abilities as target stimuli occurred in a predictable manner (every second stimulus). These two tasks had been used in a previous study showing reliable differences at behavioral and neural levels for the focus of attention and control of attention conditions, with notably an increase in task accuracy in the controlled attention condition showing that participants were able to successfully implement the controlled encoding strategy (Majerus et al., 2018).

In order to assess focus of attention abilities in the visual domain, we used the visual array task (Cowan et al., 2006; Luck & Vogel, 1997). This task presents variable amounts of information very briefly (250 ms), followed by a test item. More specifically, arrays of colored squares were presented, followed by a test array containing the same amount of squares and with one of the squares marked by a circle. The participants had to decide whether or not the color of the marked item was the same as in the target array. This task, like the running span task, yields a capacity estimation of the focus of attention of about four items (Cowan et al., 2006; Luck & Vogel, 1997). Finally, for control of attention abilities in the visual modality, a visual search task was used requiring the selection of a target visual symbol among multiple visual distractor stimuli; this task, like the auditory-verbal control of attention task, involved a strong selective attention component (Beck, Hollingworth, & Luck, 2012; Woodman & Luck, 1999). We also administered measures controlling for general non-verbal intelligence (Raven, Raven, & Court, 1998a) and processing speed (Grandjean & Collette, 2011; Salthouse & Babcock, 1991).

We predicted that verbal WM abilities should be associated with language proficiency, and this most strongly for L2 and L3, performance in L1 reaching a ceiling level. Furthermore, if attentional abilities are involved in language proficiency, then we should also observe robust associations between the different attentional measures and L2 and L3 proficiency. Of particular interest here was whether this would be the case for control of attention abilities, for which a link with language proficiency has already been demonstrated in the past in children populations (Majerus et al., 2009), and also for focus of attention abilities not yet explored in this context. Some studies have shown relationships between bilingual practice and top-down processes, namely the ability to selectively focus attention on relevant information while inhibiting irrelevant information (e.g., Krizman, Skoe, Marian, & Kraus, 2014; Rämä et al., 2018). Hence, it could be expected that the link with bilingual language proficiency might be more pronounced for control of attention as compared to focus of attention. We also determined the extent to which these associations are modality-dependent (auditory-visual vs. visuo-spatial modality) or not.

## Materials and methods

### Participants

72 participants aged 18–32 years were recruited via ads posted on social media and via a word-of-mouth procedure. Luxembourgish was the native language of one or both of their parents and was spoken on a daily basis by the participants themselves. The participants had no history of medication or drug abuse, psychiatric or neurological disorders, or learning disabilities such as dyslexia or attention deficit hyperactivity disorder (ADHD). They gave their informed consent prior to their inclusion in the study, which had been approved by the ethics committee of the University of Liège (reference number: TFE_1516-12). The participants were asked to fill in a language background questionnaire (Barbu, Gillet, Orban, & Poncelet, 2013, Unpublished questionnaire) assessing the number of languages mastered and used, linguistic family history, self-rated language use and proficiency, age and context of acquisition, and language-switching frequency. The analysis of this questionnaire revealed that for almost all participants, the dominant language was Luxembourgish. Only one participant claimed to be more fluent in German due to his professional background. Nine participants had been raised in a bilingual French-Luxembourgish family, and six had been raised in a bilingual Luxembourgish-German family. The nine participants having been raised in French-Luxembourgish families were discarded, because French could not be considered as their L3. The six participants having been raised in a bilingual German-Luxembourgish family were kept as they had reported Luxembourgish to be their dominant and most-used language. Furthermore, their formal exposure with German had started in grade one. Thus, all participants reported mastering and using Luxembourgish as their L1, German as their L2, and French as their L3. This participant selection allowed us to minimize variability due to age of acquisition and length of exposure. The data of two further participants had to be discarded due to incomplete data or because they did not fully meet the inclusion criteria (ADHD). The final sample was composed of 61 participants (34 women; mean age: 23.62 years; age range: 18–32 years; mean years of education: 14.97 years; range years of education: 12–19 years).

### Materials

#### Productive vocabulary

We assessed Luxembourgish, French and German language production with a picture-naming test containing 90 pictures (Bachy-Langedock, 1988). The test was originally developed for the French language; Luxembourgish and German translation equivalents were created for this study. The task was administered three times: the pictures had to be named once in Luxembourgish, once in German and once in French. Each picture appeared on the computer screen, and the participants were instructed to name the pictures in the target language. If they did not know the correct answer, they were instructed to say “I don’t know” before starting the next trial. The task was self-paced, and the participants scored one for each correct response. Synonyms or regional deviations of target words were tolerated. Note that some words were highly similar across the three languages (cognates). We retained the raw scores for analysis.

It could be argued that administering the same stimuli three times could induce familiarity with the pictures and thus cause a potential bias. However, it is unlikely that test exposure would influence or increase the lexicon of the participants, unless they would specifically look up unknown words between two sessions, which is, also, improbable.

#### Receptive vocabulary

We evaluated receptive vocabulary with the German and French versions of the “Peabody Picture Vocabulary Test” (Dunn & Dunn, 1959): the German PPVT (Dunn, Dunn, Bulheller, & Häcker, 2003) and the EVIP (Dunn, Thériault-Whalen, & Dunn, 1993). In these tasks, the participants are presented with four pictures for each trial. The experimenter pronounces a target word, and the participants are required to give the number of the picture which provides the best match with the word. The German version includes 89 items which are all administered to the participants in the same order. In the French version, the task starts at item 120, corresponding to the theoretical chronological age level of the participants (16+). The participant has to reach a baseline of eight correct consecutive responses, otherwise items are administered in reverse order (i.e., the items below item 120) until a baseline level of eight correct consecutive responses is attained. When the baseline is reached, the test continues after the initial item that generated an error. The test ends when the participant has reached the final item (item 170), or when a “ceiling” of at least six errors on eight consecutive trials is reached. For both the German and French versions, participant score of 1 is given for each correct response. The raw vocabulary score (sum of correct items) was retained.

#### Lexical decision

Vocabulary knowledge in German and French was further measured with the LexTALE (Brysbaert, 2013 for the French version and Lemhöfer & Broersma, 2012 for the German version), a written lexical decision task where the participants have to decide whether a given item is a word in the target language. The German version consists of 60 items (40 words and 20 nonwords) and the French version includes 84 items (56 words and 28 nonwords). The scores were obtained by calculating the percentage of correct words and correct nonwords with the following formula:

[(Number of correct words/Total number of words) × 100 + (Number of correct nonwords/Total number of nonwords) × 100]/2

An overview of these different language assessment measures is given in Table 1.

**Table 1:** Overview of the different language assessment measures.

	Reference	Description

Bachy’s Picture-naming test	Bachy-Langedock (1988)	Picture-naming test assessing productive vocabulary in Luxembourgish, French and German.
Peabody Picture Vocabulary Test (PPVT)	Dunn, Dunn, Bulheller, & Häcker (2003)	Picture-word matching test assessing receptive vocabulary in German.
Evaluation du Vocabulaire en Images Peabody (EVIP)	Dunn, Thériault-Whalen, & Dunn (1993)	Picture-word matching test assessing receptive vocabulary in French.
LexTALE German	Lemhöfer & Broersma (2012)	Lexical decision task in German.
LexTALE French	Brysbaert (2013)	Lexical decision task in French.

#### Immediate serial recall

We measured auditory-verbal WM with an immediate serial word and nonword recall task originally developed for French-speaking participants (Majerus & Van der Linden, 2003). The task was adapted into Luxembourgish for the purpose of this study. Luxembourgish one-syllable nouns were chosen, with a syllabic structure as close as possible to the original French language stimuli. As in the original task, the nonwords were identical to the words except for one phoneme. Participants heard lists of words and nonwords of growing length (one to six items) and were asked to repeat the lists in the correct serial order immediately after having heard the list. They were given the following instructions: “You are going to hear lists of words you know and lists of words that do not exist. You have to repeat these words in the same order immediately after hearing them.” The participants were informed when list length increased. For each length, there were four lists of words and four lists of nonwords. Each length always started with the recall of words, and was then followed by the recall of nonwords. There were 48 lists in total, and the maximum number of words and nonwords that could be recalled was 84 for each category. The items were presented at a rate of one stimulus/second based on a previous recording of the stimuli by a neutral female voice as .wav sound files. The participants silently listened to each list and recalled the items immediately afterwards. If the participants remembered that an item had been present in a particular position, but did not remember the item, they were asked to say “eppes” (“something”). Participants’ responses were digitally recorded for later transcription and scoring. We determined, for both word and nonword conditions, the number of items recalled in correct serial position by pooling over the different sequence lengths.

#### Focus of attention – auditory-verbal

**Figure 1:**
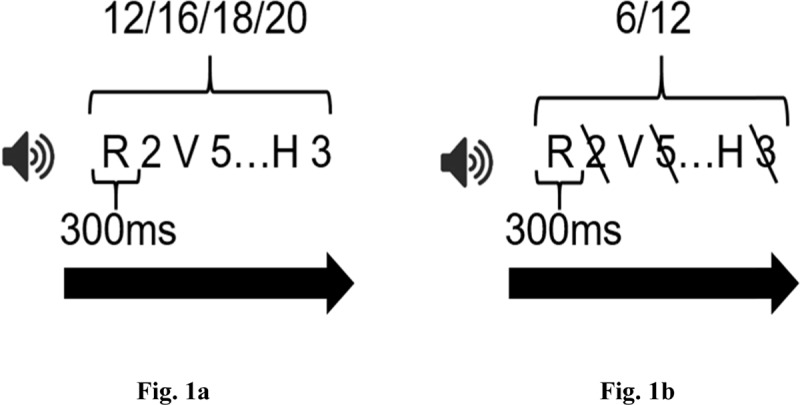
**a:** Focus of attention running span condition. Participants had to listen to every item, and recall as many items as possible in their order of presentation by starting at the end of the list. **b:** Controlled attention running span condition. Participants had to attend to only one stimulus type (for example the letters) and recall all target items in their order of presentation.

Focus of attention capacity was measured with a running span task adapted from Cowan et al.’s (2006) and translated into Luxembourgish. The participants heard lists of unpredictable length (12, 16, 18 or 20 items) pronounced at a very fast rate (2.5 items/second) (see Figure 1a). The lists consisted of digits from one to nine and of consonants, with the exception of the letter “M” because of its phonological similarity with the letter “N”, and of the letter “Q” because of its rarity of occurrence in the language. The lists always started with a letter, and responded to the pattern letter-digit-letter-digit. We excluded letter-digit combinations that might have a signification on their own and thus be chunked more easily, like “3D”. The participants listened to each list and when instructed had to recall as many items as possible from the most recent part of the list, in their order of presentation. After recall, they were invited to press the space bar to initiate the next trial. The instructions were as follows: “You will hear rapid sequences of digits and letters. Listen to them carefully since you will have to report as many digits and letters as possible when a sequence stops. Be careful, you will need to recall them in the correct order from the end of the sequence.” The task consisted of five training trials and 20 test trials. Task presentation was controlled via E-prime 2.0 (https://pstnet.com/welcome-to-e-prime-2-0/) software based on digital recordings of the auditory stimuli by a neutral female voice. The participants’ output was recorded for later transcription and scoring. The total number of items reported in correct serial order (from the end of the list) was retained.

#### Controlled attention – auditory-verbal

The items were the same as in the focus of attention condition, namely digits and consonants, presented in a letter-digit-letter-digit continuous sequence (see Figure 1b). List length varied between 6 and 12 items and were presented in random order. Participants were instructed to focus specifically on the letters in one half of the trials and on the digits in the other half, and to ignore the non-target category. At the end of each list, they had to report as many target items as possible in their order of presentation, starting at the beginning of each list. Like for the focus of attention task, the participants had to press the space bar to initiate the next trial. The task instructions were the following: “You will hear rapid sequences of digits and letters. Listen to them carefully since you will have to report as many digits or letters as possible in the correct serial order when a sequence stops. The test will be divided into two parts. In the first part, you will have to focus only on the digits/letters and recall only the digits/letters. In the second part, you will have to focus only on the letters/digits and recall only the letters/digits.”

There were five training trials and 10 trials per target stimulus type. The order of administration of conditions was counterbalanced across participants. Task presentation and scoring was identical to the previous task.

#### Focus of attention – visuo-spatial

**Figure 2:**
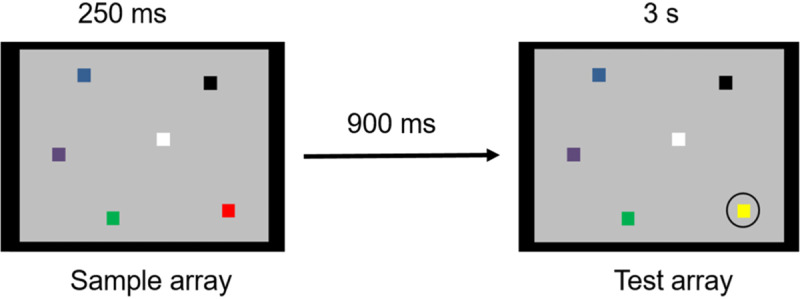
Sample and test arrays for the visuo-spatial focus of attention task.

Passive focus of attention capacity in the visuo-spatial modality was assessed with a visual sequential comparison task adapted from Luck and Vogel (1997) where participants had to indicate whether two visual arrays were identical or different. Participants visualized a sample array of two, four, six or eight colored squares that briefly appeared on screen. After a short delay of 900 ms, a test array appeared, and participants had to determine if the color of a target square, surrounded by a circle, was identical to the color in the sample array (see Figure 2). The color of the non-target squares in the array did not change. They received the following instructions: “You will see an array of two, four, six or eight squares of different colors. You will then see a second array with squares positioned in the same locations as in the first array. In the second array, a square will be circled. You will have to decide whether the color of the circled square is the same as the color of the square located at the same place in the first array. Push the “Yes”- button if the color is the same (the color of the square has not changed), and push the “No”-button if the color is not the same (the color of the square has changed). Please respond as quickly as possible.”

The trials started with a fixation cross that was displayed on a grey 13.31° × 10° screen for 750 ms. The sample array appeared for 250 ms. The squares were presented on a grey background and had a size of 0.67° × 0.67°, and their colors were highly distinguishable (yellow, blue, green, violet, red, black and white). The sample and test array were separated by a 900-ms interval, during which the screen remained grey. The test array remained on screen for three seconds. If the participants had not produced a response within this time window, the next trial immediately started. There were four training and 64 test trials including 33 identical and 31 dissimilar arrays. Note that the participants could repeat the training trials until they felt at ease with the task. Task performance was estimated by the *k*-index (Cowan et al., 2005), which gives a measure of the amount of items held in the focus of attention corrected for guessing responses. The formula is the following:

{\rm{k}} = {\rm{N}}*\left( {{\rm{H}} + {\rm{CR}}\ -\ 1} \right),

with N being the number of items presented in the array, H the proportion of Hits and CR the proportion of correct rejections. For each participant, the focus of attention was defined as the highest k value.

#### Controlled attention

**Figure 3:**
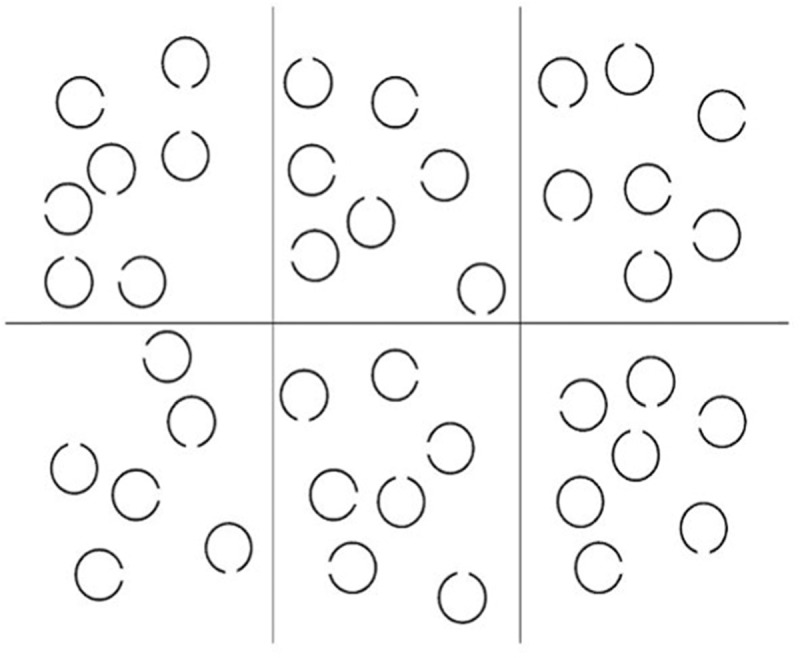
Visual controlled attention task. The participants were presented with an array of circles distributed over six rectangles, and had to find as quickly as possible the rectangle containing the target item (here the full circle).

Controlled attention in the visuo-spatial modality was measured with a visual search task adapted from Woodman and Luck (1999). The task consists of an array of 41 black circles (full circles and circles with a small opening at the top, at the bottom, on the right side or on the left side) on a white background distributed over six rectangles containing each six or seven circles. The circles had a diameter of 1.43° and an opening of 0.48°. Each rectangle had a dimension of 8.58° × 9.05°, and the entire background containing the six rectangles had a dimension of 24.27° × 18.46°. The experiment was divided into five blocks, where participants had to find a target circle that was present only once in the array (see Figure 3). In each block, another type of circle (full circle or one of the four open circles) had to be detected. The target circle was presented prior to each trial block. The participants were asked to press a button representing the rectangle in which the target item was situated. The instructions were the following: “Five types of circles are going to be presented on the screen (full circles and circles with a small opening at the top, at the bottom, on the right side or on the left side). This task will be divided into five parts. In every part, an array of circles will appear on the screen. You will have to detect as quickly as possible the target circle type among the four other types of circles. The target circle type will be indicated at the beginning of each part. The computer screen will be divided into six sections. You will respond by selecting the response button (numbers from one to six) on the numeric keypad corresponding to the number of the section in which the target circle is located.”

The bottom sections were numbered one, two and three (from left to right), and the top sections were numbered four, five and six (from left to right), imitating the key placements of the numeric keypad. Each of the five blocks contained one training trial and 12 test trials. The array stayed on screen for nine seconds. If the participants had not found the target item within this time window, the next trial started automatically. The final score was defined as the proportion of correct trials across the whole task.

#### Intellectual efficiency

Non-verbal intellectual efficiency was estimated using Raven’s standard progressive matrices (Raven et al., 1998a). Participants were given 20 minutes to complete the task. We retained the raw scores.

#### Processing speed

Processing speed was evaluated with a task adapted from Salthouse and Babcock (1991) and Grandjean and Collette (2011) for the computerized version. In this task, the participants viewed two capital letters written in white on a black screen, and had to determine as fast as possible whether these letters were identical or dissimilar. There were 6 training and 64 test trials. The dependent variable was the mean response time for correct trials only.

### Task order

The experiment was conducted in two sessions lasting 1.5 to 2 hours each and taking place in a quiet room. In each session, a break was allowed after half of the tests were completed. The two LexTALE tasks and Raven’s matrices were conducted on paper sheets. All other tests were presented via a Dell Latitude E5420 Essential (14 inches) or a Dell Latitude E5570 (15 inches) laptop. Participants were comfortably seated approximately 60 cm away from the computer screen. Auditory stimuli were presented via headphones connected to the laptop. The tasks were administered in three different, counterbalanced orders.

### Data analysis

A Bayesian statistical approach was used. Bayesian statistics have the advantage, relative to frequentist statistics, to determine the strength of the evidence both against and in favor of the null hypothesis in order to identify which effect is associated with the strongest evidence (Clark et al., 2018; Kruschke, 2010; Lee & Wagenmakers, 2013; Nuijten, Wetzels, Matzke, Dolan, & Wagenmakers, 2015; Wagenmakers et al., 2018). Bayesian statistics also allow for multiple statistical tests to be carried out without increasing first type error risk (Clark et al., 2018). The Bayes Factor (BF) is the likelihood ratio of a given model, the best-fitting model being the one with the highest BF. BF_01_ indicates evidence in favor of the null hypothesis, while BF_10_ indicates evidence in favor of the alternative hypothesis. Although there are no fixed thresholds for BF values, we used the following categories for describing strength of evidence: a BF of at least 3 is considered to indicate moderate evidence, a BF of at least 10 is considered to provide strong evidence, a BF of at least 30 is considered to provide very strong evidence, and a BF of at least 100 is considered to indicate decisive evidence (Jeffreys, 1998). Bayesian correlation analyses were performed using the JASP statistical package with default prior settings (JASP team, 2019, Version 0.11.1). Partial Bayesian correlations were conducted using the JZS method (Jarosz & Wiley, 2014; Rouder, Speckman, Sun, Morey, & Iverson, 2009) from the Bayes Med package (Nuijten et al., 2015) in R (R core team, 2019). This analysis allowed us to investigate the specificity of the link between language proficiency and WM measures while controlling for intellectual efficiency, age, and processing speed. Additional Bayesian regression analyses are also reported in supplementary material S1–S3.

Sensitivity of a correlational Bayesian statistical design (the equivalent of power analysis for frequentist analyses) was assessed with BFDA package implemented in R (Schönbrodt & Stefan, 2018). The indicative effect size for these analyses was of r = .30 based on previous studies investigating the links between verbal WM and lexical language proficiency (Gathercole et al., 1994; Majerus & Boukebza, 2013; Majerus et al., 2008; Ordonez Magro et al., 2018). This analysis showed that if the correlation of interest existed, the minimal sample size needed for reaching minimal evidence (BF_10_ > 3) in favor of the effect in 80% of simulated samples was N = 60.

We further calculated split-half reliabilities for all tests. We divided the tasks into two equal halves by alternatively assigning items to one or the other test half, using a procedure similar to Lemhöfer and Broersma (2012). The scores for the two halves were correlated over all participants and a further correction for test length as proposed by Frey (2018) was applied. Reliability values are displayed in Table 2.

**Table 2:** Descriptive statistics and reliability measures.

	Mean	Std. Deviation	Minimum	Maximum	Split-half reliability

Age	23.623	3.050	18.000	32.000	
Education	14.967	2.041	12.000	19.000	
AoA L2	5.492	1.850	0.000	7.000	
AoA L3	7.246	0.434	7.000	8.000	
Naming L1	82.164	3.675	72.000	89.000	0.651
Naming L2	78.607	5.011	61.000	87.000	0.738
Naming L3	54.180	8.186	37.000	71.000	0.878
PPVT (L2) (raw score)	71.918	6.176	54.000	84.000	0.806
EVIP (L3) (raw score)	135.098	15.951	106.000	166.000	0.963
LexTALE L2	83.832	7.158	61.250	100.000	0.660
LexTALE L3	68.970	9.937	43.750	90.179	0.863
ISR words	66.098	8.491	44.000	78.000	0.789
ISR nonwords	40.361	7.543	23.000	60.000	0.789
FoA – auditory-verbal	52.705	13.020	3.000	79.000	0.811
CoA – auditory verbal	42.918	12.272	10.000	70.000	0.807
FoA – visuo-spatial	3.904	1.583	1.000	8.000	0.639
CoA – visuo-spatial	0.716	0.105	0.433	0.900	0.797
Raven’s (raw score)	50.328	4.430	39.000	59.000	0.757
PS	588.713	68.970	434.967	796.672	0.950

*Note*: AoA = Age of acquisition; ISR = immediate serial recall; FoA = focus of attention; CoA = control of attention; Raven’s = Raven’s matrices; PS = processing speed.

## Results

### Descriptive statistics

Descriptive statistics of all measures are displayed in Table 2. All further analyses were conducted using standardized scores given that the range of scores strongly differed between tasks. As shown in Table 2, L2 was formally acquired at six or seven years of age (i.e., first grade) for all participants. As already mentioned, six participants had however been raised in bilingual Luxembourgish-German families and had been exposed to L2 before formal exposure. All participants had formally acquired L3 at seven or eight years of age (i.e., second grade). Naming in L3 led to lower scores than naming in L2 (Bayesian Paired T-Test: BF_10_ = 1.198e+25) and L1 (Bayesian Paired T-Test: BF_10_ = 7.789e+25) which was indeed expected given that French (L3) is the latest acquired language and differs at phonological and lexical levels from L1 to a greater extent than L2 (German). L1 also led to better naming performance as compared to L2 (Bayesian Paired T-Test: BF_10_ = 17488). This analysis confirmed the results from the language background questionnaire indicating that Luxembourgish was the first and best-mastered language, followed by German and French. Most reliability estimates were of moderate to large size, except for slightly smaller values for the L1 naming, L2 lexical decision, and visuo-spatial focus of attention measures.

### Correlation analyses

First, we determined the correlations between the different language measures (i.e., naming, receptive vocabulary and lexical decision) in order to further assess their reliability (see Table 3). We observed that within a given language, the tasks all showed strong associations. Interestingly, naming in L1 was moderately to decisively associated with all L2 measures, which might stem from the similarities between Luxembourgish and German. Furthermore, L1 naming was strongly associated with L3 naming, which suggests the existence of common processes between naming abilities in all languages.

**Table 3:** Bayesian Pearson Correlations.

	1	2	3	4	5	6

1. Naming L1	—					
2. Naming L2	**0.694**^2.267e +7^	—				
3. PPVT	**0.448**^95.474^	**0.544**^3676.036^	—			
4. LexTALE L2	**0.325**^3.874^	**0.452**^110.015^	**0.430**^54.803^	—		
5. Naming L3	**0.393**^19.497^	0.235^0.810^	0.143^0.288^	0.093^0.205^	—	
6. EVIP	0.116^0.235^	0.112^0.229^	0.267^1.323^	0.302^2.451^	**0.457**^126.858^	—
7. LexTALE L3	0.042^0.168^	–0.045^0.169^	–0.017^0.161^	–0.092^0.204^	**0.762**^9.504e +9^	**0.406**^27.379^

*Note*: The exponents represent the BF values. The correlations associated with a BF value of at least 3 are in bold.

Next, we determined the correlations between language proficiency, WM and attentional abilities while also entering Raven’s matrices, processing speed and age. For this analysis, we calculated a general language proficiency index based on the mean of the standardized scores in each test for a given language. The full correlation matrix and BF values are given in Table 4. Correlations between L1, L2 and L3 language proficiency measures and WM measures were associated with moderate to very strong Bayesian evidence, only for the nonword measure for L1 and for both word and nonword measures for L2 and L3. Both auditory-verbal control of attention and focus of attention measures showed also robust associations with the word and nonword WM measures. However, contrary to our predictions, none of the attentional measures correlated with L2 and L3 proficiency measures. The visuo-spatial attention measures did not show any robust association with any language or WM measure. For visual focus of attention, the strongest correlation was observed with Raven’s matrices, while for controlled visual attention, the strongest correlation was found with processing speed.

**Table 4:** Bayesian Pearson Correlations.

	1	2	3	4	5	6	7	8	9	10	11

1. Naming L1	—										
2. Language Proficiency L2	**0.606**^77114.981^	—									
3. Language Proficiency L3	0.220^0.663^	0.165^0.352^	—								
4. ISR words	0.273^1.473^	**0.336**^4.903^	**0.435**^63.046^	—							
5. ISR nonwords	**0.322**^3.636^	**0.326**^3.997^	**0.364**^9.249^	**0.747**^2.138e +9^	—						
6. FoA – auditory-verbal	0.182^0.418^	0.238^0.842^	0.287^1.855^	**0.484**^322.723^	**0.467**^179.695^	—					
7. CoA – auditory-verbal	0.179^0.404^	0.216^0.626^	0.252^1.041^	**0.475**^239.890^	**0.529**^1923.114^	0.278^1.578^	—				
8. FoA – visuo-spatial	–0.012^0.160^	0.187^0.440^	0.093^0.205^	0.191^0.462^	0.015^0.161^	–0.100^0.214^	0.003^0.160^	—			
9. CoA – visuo-spatial	0.164^0.347^	0.125^0.251^	–0.069^0.183^	0.194^0.480^	0.131^0.262^	0.150^0.307^	0.113^0.231^	–0.011^0.160^	—		
10. Age	0.095^0.207^	0.299^2.310^	0.071^0.185^	0.088^0.200^	0.055^0.174^	0.118^0.239^	–0.020^0.162^	0.093^0.204^	0.041^0.168^	—	
11. Raven’s	–0.000^0.160^	0.304^2.566^	0.091^0.203^	0.308^2.778^	0.197^0.495^	0.177^0.395^	0.282^1.703^	0.304^2.533^	0.249^0.994^	0.073^0.187^	—
12. Processing speed	0.230^0.752^	0.023^0.162^	0.094^0.206^	–0.177^0.398^	–0.062^0.179^	–0.201^0.516^	–0.090^0.202^	0.112^0.229^	**–0.350**^6.737^	0.190^0.457^	–0.308^2.735^

*Note*: The language proficiency score represents the mean of the standardized scores in each test for a given language. For all auditory-verbal tasks, the score displayed represents the number of items recalled in the correct serial order. For the visuo-spatial tasks, the focus of attention was measured with the k estimate, and control of attention was measured as the proportion of correctly identified items. The exponents represent the BF values. The correlations associated with a BF value of at least 3 are in bold. ISR = immediate serial recall; FoA = focus of attention; CoA = control of attention; Raven’s = Raven’s matrices.

In order to assess the specificity of the observed correlations, we conducted partial correlations between the serial recall measures and L1–L3 proficiency controlling for intellectual efficiency, age and processing speed. As shown in Table 5, the correlation between L1 proficiency and word recall was associated with moderate evidence when partialling out processing speed, but became anecdotal when partialling out Raven’s matrices and age. Nonword recall remained moderately associated with L1 when partialling out the three control variables. For L2, while the evidence for the association between word/nonword recall and proficiency remained moderate when partialling out age and processing speed, it became anecdotal when partialling out Raven’s matrices for both measures. For L3, the evidence for all associations remained moderate to decisive after controlling for intellectual efficiency, processing speed and age. This indicates that the most reliable link between language ability and WM abilities is observed for L3, the latest learned and not yet fully mastered language.

**Table 5:** Bayesian Partial Correlations.

Naming L1			

WM measures	Control variables	*r*	BF_10_

ISR words	Raven’s	0.288	1.768
ISR words	Age	0.267	1.299
ISR words	PS	0.328	**4.006**
ISR nonwords	Raven’s	0.328	**4.022**
ISR nonwords	Age	0.319	**3.341**
ISR nonwords	PS	0.346	**6.011**
**Language Proficiency L2**			

**WM measures**	**Control variables**	***r***	**BF**_10_

ISR words	Raven’s	0.267	1.247
ISR words	Age	0.326	**3.863**
ISR words	PS	0.346	**5.879**
ISR nonwords	Raven’s	0.285	1.746
ISR nonwords	Age	0.325	**3.849**
ISR nonwords	PS	0.329	**4.105**
**Language Proficiency L3**			

**WM measures**	**Control variables**	***r***	**BF**_10_

ISR words	Raven’s	0.429	**50.597**
ISR words	Age	0.431	**55.949**
ISR words	PS	0.461	**141.753**
ISR nonwords	Raven’s	0.354	**7.155**
ISR nonwords	Age	0.361	**8.594**
ISR nonwords	PS	0.372	**11.129**

*Note*: BF values of at least 3 are in bold. ISR = immediate serial recall; Raven’s = Raven’s matrices; PS = Processing speed.

## Discussion

The present exploratory study aimed at investigating the links between WM, attention and language proficiency in adult trilingual speakers. Using a Bayesian correlation approach, we observed moderate to strong associations between verbal WM abilities and language proficiency in L3, and to a slightly lesser extent in L2 and L1, in line with our predictions. At the same time, no robust association was observed between auditory-verbal and visuo-spatial attention abilities and language proficiency.

On the one hand, the results support and extend previous findings arguing for an association between auditory-verbal WM abilities and non-native language learning (Cheung, 1996; Majerus et al., 2008; Service, 1992). In line with this hypothesis, we observed the strongest association between both auditory-verbal WM measures and language proficiency in L3 whose mastery was lower as compared to the other languages, and for which learning processes are still ongoing (Gathercole & Masoura, 2005, Service, 1992). Our results therefore support the continuous role of WM in language abilities throughout adulthood. At the same time, for native language as well as non-native languages mastered with high proficiency, the link with WM might progressively fade, and further language proficiency might rely more on environmental variables such as amount of language use and exposure which steadily increase as one gets older (Barbosa, Jiang, & Nicoladis, 2019; Gathercole & Masoura, 2005); the latter interpretation is also in line with the association observed between L2 proficiency and chronological age. It should be noted here that the split-half reliabilities for some L1 and L2 measures were slightly lower than for those in L3. Hence, caution is needed when comparing the associations between the language and WM measures as a function of language type. At the same time, the robust correlations between the different language tests suggest that the measures were reliable and tapping into similar abilities. It should also be noted that L1 was assessed only via a picture naming test. It is also interesting to note that L1 and L2 proficiency were highly correlated, with smaller correlations between L1 and L3 as we may expect based on the language families involved here. Luxembourgish (L1) and German (L2) are indeed both Germanic languages, sharing a large number of features at the phonological, grammatical and lexical level. On the other hand, French (L3) is a Romance language more distant from Luxembourgish at phonological and grammatical levels, and hence may be more difficult to acquire for a native speaker of Luxembourgish. For the same reason, language proficiency in that case may also be linked more strongly with verbal WM abilities as these will be solicited more extensively during the language learning process. Future studies might further explore this question by assessing multilingual populations involving languages from different families (e.g., Spanish versus Italian or versus Arabic). In sum, the results observed in this study are in line with theoretical accounts considering that verbal WM plays a role in language learning, at least as regards lexical aspects of language learning (Baddeley et al., 1998; Gupta & MacWhinney, 1997; Majerus, Poncelet, Elsen, et al., 2006; Majerus, Poncelet, Greffe, et al., 2006), although note that we did not directly measure language learning, but rather its consequence in the form of language proficiency at a given point of time.

Note that the associations between language proficiency and verbal WM could at least partly be related to the fact that L2 and L3 were mainly acquired in a school context. Indeed, vocabulary is often taught by associating target words to their equivalents in the native language or to pictures. This learning method shares similarities with paired-associate learning, which has been shown to be strongly associated with verbal WM, especially WM for serial order (e.g., Majerus & Boukebza, 2013; Majerus et al., 2008; Majerus, Poncelet, Elsen, et al., 2006). At the same time, although formal learning takes place mainly in an academic context, German and French are also used in social and professional contexts outside the educational setting in Luxembourg. Hence, the associations observed between WM and language knowledge may not be solely determined by the academic nature of L2 and L3 learning in Luxembourg.

So far, we have interpreted the associations observed between verbal WM and language proficiency as supporting a determining role of WM in language proficiency. We however need to consider a further alternative interpretation of our results, namely that it is the multilingual context that leads to improved working memory abilities, as mentioned earlier (Adesope et al., 2010; Bialystok, 2009, 2011, 2015; Bialystok et al., 2004). It follows that, if the ‘bilingual cognitive advantage’ hypothesis was to explain the associations observed between verbal WM abilities and L2/L3 language proficiency in the present study, then we should also have expected an association between L2/L3 language proficiency and the most ‘executive’ measures used in this study, namely the controlled attention measures. This type of measures has indeed been most consistently associated with bilingual cognitive advantages (e.g., Bialystok, 2011). As we will discuss in the next paragraph, this was not the case.

An intriguing finding of this study is indeed the lack of reliable evidence for an association between language proficiency and attentional abilities in both auditory-verbal and visuo-spatial domains. We had predicted that particularly auditory-verbal attention abilities should be associated with language proficiency given their association with the auditory-verbal WM tasks. Our hypothesis was based on past studies (Gomes et al., 2007; Majerus et al., 2009; Montgomery et al., 2009) that had observed that auditory-verbal attention supports verbal WM and language acquisition in typically developing children (Majerus et al., 2009), in children with selective language impairment (Montgomery et al., 2009), and in children with various behavioral or reading problems (Gomes et al., 2007). We should note here that the studies on which our hypothesis was based recruited exclusively children populations while the present study focused on young adult participants. Whereas bilingual language proficiency still appears to be influenced to a significant extent by auditory-verbal WM abilities in adult populations, the results of the present study suggest that attentional abilities, involving high or low attentional control demands in the form of control of attention and focus of attention tasks, do not contribute (anymore) to language proficiency, at least as regards the mainly lexical aspects of language abilities that were assessed in this study. It may still be that attentional control abilities contribute to non-native language learning but that these demands are relatively minimal and easily met by the attentional abilities for already established language representations in adult participants. It remains to be shown whether a more direct measure of language learning may have revealed more reliable associations with attentional abilities. Note that although the association was anecdotal, the evidence in favor of a reliable correlation between language proficiency and focus of attention was strongest for L3, which suggests that some attentional processes might be involved in language ability for languages mastered with lower levels of proficiency.

A further interesting finding of this study is the observation of a strong association between the verbal WM and the auditory-verbal attention measures but not between verbal WM and the visuo-spatial attention measures. There is currently a debate about the domain-specificity of attentional resources involved in WM. On the one hand, a number of studies suggest that attentional processes involved in WM tasks are domain-general (e.g., Cowan et al., 2011; Majerus et al., 2016) while other studies observe distinct capacity estimates and neural substrates for auditory-verbal versus visuo-spatial attention abilities involved in WM tasks (Chen & Mitra, 2009; Majerus et al., 2018; Morey & Cowan, 2005; Morey & Miron, 2016). The results of the present study appear to support the existence of modality-specific attentional abilities, and this both for focus of attention and control of attention components. It could be argued that the auditory-verbal attention tasks were structurally more similar to the WM task used in this study than were the visuo-spatial attention tasks (see also below). However, if the structural similarity of the tasks is exclusively responsible for the association observed between the auditory-verbal attention and WM tasks, then the auditory-verbal attention tasks should have shown the same associations with language proficiency indexes as did the WM tasks, which was not the case.

At the same time, we need to acknowledge several limits of this study. A first limit is that we were unable to control for the lexical frequency of the Luxembourgish stimuli used in the different tasks of this study given that, to our knowledge, there is no lexical database for the Luxembourgish language. The construction of the Luxembourgish stimuli was based on translation equivalents from the original French stimuli for which lexical frequency could be controlled. Given that the Luxembourgish language context is of particularly high interest for the study of multilingualism, and particularly trilingualism, future studies focusing on the development of lexical databases for the Luxembourgish language are clearly needed. A further limitation of this study is the difficulty to obtain an objective and direct measure of the amount of native and non-native language exposure. Although great care was taken in selecting participants with similar ages of acquisition of L2 and L3 and similar duration of exposure to L2 and L3, when being adults, the amount of exposure to the different languages might still differ between participants, depending on specific living and professional language contexts. Another limitation concerns the structural differences between the visuo-spatial and auditory-verbal attentional measures which have already been mentioned above. The visuo-spatial controlled attention task required attentional control driven by a single target item, while the three other tasks required focus/control of attention for a larger set of items. These structural differences urge us to remain cautious in our interpretations about the role of attention in WM and language learning, and they may also explain why there was no reliable correlation between the visuo-spatial focus and control of attention tasks. Note also that the split-half reliability for the visuo-spatial focus of attention task was lower than for the other tasks, which might have further contributed to the lack of association between the two visuo-spatial attention tasks. At the same time, it should be noted that the tasks used in these studies are those that are commonly used to measure focus and control of attention in auditory-verbal and visual domains (e.g., Anderson, Vogel, & Awh, 2013; Cowan et al., 2005, 2006). We should also acknowledge that the results of this correlational study do not directly inform us about the direction of the associations observed between WM and language proficiency; longitudinal studies would be needed to tease apart the relative contribution of WM to language proficiency, and of language proficiency to WM. Finally, at a statistical level, it could be considered that the use of a default continuous uniform distribution for the prior in correlation analyses using the Bayesian framework may be considered suboptimal or even implausible as this prior considers that the probability of the correlation falling into one interval of all possible correlation values (ranging from –1 to 1) will be equal to the probability of the observation falling into any other interval of the same size. Also, the Bayesian approach being more conservative than a frequentist approach, we need to be cautious when interpreting the results, especially as regards evidence for the null (Brysbaert, 2019). Note however that when we ran all analyses using an inverted U-shape prior distribution with the mass centered on zero, the outcome of results did not change in a significant manner, except for the results becoming slightly less conservative.

## Conclusion

The present study provides novel evidence for a strong association between auditory-verbal WM abilities and non-native language proficiency for languages not yet fully mastered. Importantly, our results indicate that this association cannot be accounted for by the auditory-verbal attentional requirements of verbal WM tasks.

## Additional File

The additional file for this article can be found as follows:

10.5334/pb.525.s1 Supplementary Material. Multiple regression analyses.
