# Supplementary material for: Verbal Working Memory but Not Attention Is Related to Language Proficiency: Evidence from Multilingual Speakers
Source: Psychol Belg. 2020 Sep 4;60(1):270–93. doi: 10.5334/pb.525 (PMC7473201; doi:10.5334/pb.525)
Supplement: Supplementary Material. — Multiple regression analyses. [file pb-60-1-525-s1.pdf]

Supplementary Material : Multiple regression analyses

Additional regression analyses assessed the specificity of the prediction of language proficiency scores by the word/nonword WM scores after including Raven's matrices, age and processing speed in the null model. The same type of analyses were also conducted for auditory-verbal and visuo-spatial attentional predictor measures. The results for the first set of Bayesian regression analyses with the word/nonword WM predictor measures is displayed in Table S1. For the L1 proficiency score, the model with the highest BF_{10} value included the nonword recall measure (R^2 = 0.167; BF_{10} = 6.725). This model was 1.39 more likely than the model including word recall. For the L2 proficiency score, no model was associated with a BF_{10} above 3; the model associated with the strongest evidence included the nonword recall measure (R^2 = 0.242; BF_{10} = 2.933), showing only very moderate evidence in favor of an association between L2 and nonword recall. Hence, when including age, processing speed and Raven's matrices to the null model, the association between L2 and nonword recall is no more stronger than the association between L1 and nonword recall. Note however that this observation might be due to model overdetermination. Finally, for the L3 proficiency score, the model associated with the strongest evidence included word recall (R^2 = 0.219; BF_{10} = 65.223). This model was 2.28 times more likely than the model including both word and nonword recall and should be retained as the more parsimonious model. In sum, the nonword WM score was the strongest specific predictor of the least well mastered language (L3), while the word WM score was the strongest specific predictor of both L2 and L1 proficiency.

<u>Table S1.</u> Bayesian regression including L1 (Luxembourgish), L2 (German), and L3 (French) as dependent variable and word/nonword recall score as independent variables. Raven's matrices, processing speed and age have been included in the null model.

**L1 : ISR**					
**Models**	**P(M)**	**P(M|data)**	**BF_M**	**BF_{10}**	**R^2**
Null model (incl. Age. PS. Raven's)	0.333	0.094	0.207	1.000	0.060

ISR nonwords	0.167	0.315	2.301	6.725	0.167
ISR words	0.167	0.228	1.474	4.858	0.155
ISR words + ISR nonwords	0.333	0.364	1.142	3.879	0.177

**L2 : ISR**					
**Models**	**P(M)**	**P(M|data)**	**BF_M**	**BF_{10}**	**R^2**
Null model (incl. Age. PS. Raven's)	0.333	0.200	0.500	1.000	0.173
ISR nonwords	0.167	0.293	2.074	2.933	0.242
ISR words	0.167	0.225	1.449	2.247	0.233
ISR words + ISR nonwords	0.333	0.282	0.786	1.411	0.247

**L3 : ISR**					
**Models**	**P(M)**	**P(M|data)**	**BF_M**	**BF_{10}**	**R^2**
Null model (incl. Age. PS. Raven's)	0.333	0.015	0.030	1.000	0.026
ISR words	0.167	0.487	4.756	65.223	0.219
ISR words + ISR nonwords	0.333	0.427	1.492	28.585	0.221
ISR nonwords	0.167	0.070	0.378	9.402	0.150

Note. PS = Processing speed; Raven's = Raven's matrices.

The next set of regression analyses assessed the prediction of language proficiency scores by the auditory-verbal attentional measures (focus of attention and control of attention tasks) while including Raven's matrices, age and processing speed in the null model. The results of this analysis are shown in Table S2. For L1, no model was associated with a BF_{10} above 3. The model associated with the strongest evidence included focus of attention (R^2 = 0.109; BF_{10} = 1.492). Similarly, for L2, the model associated with the strongest evidence included the focus of attention factor (R^2 = 0.203; BF_{10} = 0.928). Finally, for L3, the model associated with the strongest evidence was focus of attention (R^2 = 0.114; BF_{10} = 3.748). This model was only 1.05 times more likely than the model including also controlled attention, but should be retained as the most parsimonious model. Thus, when including Raven's matrices, processing speed and age in the null model, an association between L3 and focus of attention can be observed but at the same time no reliable raw correlation was observed between these

variables, or between L3 proficiency and the control variables. Hence, these results have to be interpreted with caution.

Table S2. Bayesian regression including L1 (Luxembourgish), L2 (German), and L3 (French) as dependent variables and the auditory-verbal focus of attention and control of attention tasks. Raven's matrices, processing speed and age have been included in the null model.

**L1: Auditory-verbal attention**					
**Models**	**P(M)**	**P(M|data)**	**BF_M**	**BF_{10}**	**R^2**
Null model (incl. Age, PS, Raven)	0.333	0.293	0.828	1.000	0.060
FoA	0.167	0.219	1.398	1.492	0.109
FoA + CoA	0.333	0.330	0.986	1.127	0.128
CoA	0.167	0.158	0.941	1.082	0.096
**L2 : Auditory-verbal attention**					
**Models**	**P(M)**	**P(M|data)**	**BF_M**	**BF_{10}**	**R^2**
Null model (incl. Age, PS, Raven)	0.333	0.420	1.451	1.000	0.173
FoA	0.167	0.195	1.211	0.928	0.203
CoA	0.167	0.155	0.917	0.737	0.195
FoA + CoA	0.333	0.230	0.596	0.546	0.215
**L3 : Auditory-verbal attention**					
**Models**	**P(M)**	**P(M|data)**	**BF_M**	**BF_{10}**	**R^2**
Null model (incl. Age, PS, Raven)	0.333	0.137	0.317	1.000	0.026
FoA	0.167	0.256	1.721	3.748	0.114
FoA + CoA	0.333	0.487	1.902	3.567	0.143
CoA	0.167	0.120	0.681	1.754	0.083

Note. FoA = focus of attention; CoA = control of attention; Raven's = Raven's matrices; PS = processing speed.

The third set of regression analyses followed the same logic but included the visuo-spatial attention measures as predictor measures instead of the auditory-verbal attention measures. The results of this analysis are displayed in Table S3. Given the overall low BF_{10} values, we also computed the BF_{01} in order to assess the evidence against the alternative hypothesis. For L1, the model associated with the highest BF_{10} value included the controlled attention predictor (R^2 = 0.122; BF_{10} = 2.072), while the model associated with the strongest

evidence in favor of the null hypothesis included the focus of attention (R^2 = 0.065; BF_{01} = 1.921). Thus, for L1, the models were neither clearly in favor of the alternative hypothesis, nor against it. For L2, the model associated with the strongest BF_{10} value included the focus attention and control of attention predictors, both associated with the same values (R^2 = 0.178; BF_{10} = 0.454). In turn, the model associated with the highest BF_{01} value included both focus of attention and controlled attention predictors (R^2 = 0.182; BF_{01} = 4.401). Similarly, for L3, the model associated with the strongest evidence in favor of the alternative hypothesis included the control of attention measure (R^2 = 0.030, BF_{10} = 0.528) and the model associated with the strongest evidence in favor of the null hypothesis included both attentional measures (R^2 = 0.032; BF_{01} = 3.527). In sum, reliable evidence against an association between language proficiency and visuo-spatial attention abilities was observed for L2 and L3.

Table S3. Bayesian regression including L1 (Luxembourgish), L2 (German), and L3 (French) as dependent variables and the visuo-spatial focus of attention and control of attention tasks. Raven's matrices, processing speed and age as independent variables. Raven's matrices, processing speed and age have been included in the null model.

**L1 : Visuo-spatial attention**						
**Models**	**P(M)**	**P(M|data)**	**BF_M**	**BF_{10}**	**BF_{01}**	**R^2**
Null model (incl. Age, PS, Raven's)	0.333	0.297	0.844	1.000	1.000	0.060
CoA	0.167	0.307	2.219	2.072	0.483	0.122
FoA + CoA	0.333	0.319	0.935	1.074	0.931	0.126
FoA	0.167	0.077	0.418	0.521	1.921	0.065

**L2 : Visuo-spatial attention**						
**Models**	**P(M)**	**P(M|data)**	**BF_M**	**BF_{10}**	**BF_{01}**	**R^2**
Null model (incl. Age, PS, Raven's)	0.333	0.595	2.936	1.000	1.000	0.173
CoA	0.167	0.135	0.781	0.454	2.202	0.178
FoA	0.167	0.135	0.780	0.454	2.205	0.178
FoA + CoA	0.333	0.135	0.313	0.227	4.401	0.182

L3 : Visuo-spatial attention

Models	P(M)	P(M|data)	BF_M	BF_{10}	BF_{01}	R^2
Null model (incl. Age, PS, Raven's)	0.333	0.556	2.506	1.000	1.000	0.026
CoA	0.167	0.147	0.861	0.528	1.893	0.030
FoA	0.167	0.139	0.809	0.501	1.996	0.028
FoA + CoA	0.333	0.158	0.374	0.284	3.527	0.032

Note. FoA = focus of attention; CoA = control of attention; Raven’s = Raven’s matrices; PS = processing speed.
